# MicroCellClust: mining rare and highly specific subpopulations from single-cell expression data

**DOI:** 10.1093/bioinformatics/btab239

**Published:** 2021-04-08

**Authors:** Alexander Gerniers, Orian Bricard, Pierre Dupont

**Affiliations:** ICTEAM/INGI/Artificial Intelligence and Algorithms Group, UCLouvain, Louvain-la-Neuve 1348, Belgium; de Duve Institute, UCLouvain, Brussels 1200, Belgium; ICTEAM/INGI/Artificial Intelligence and Algorithms Group, UCLouvain, Louvain-la-Neuve 1348, Belgium

## Abstract

**Motivation:**

Identifying rare subpopulations of cells is a critical step in order to extract knowledge from single-cell expression data, especially when the available data is limited and rare subpopulations only contain a few cells. In this paper, we present a data mining method to identify small subpopulations of cells that present highly specific expression profiles. This objective is formalized as a constrained optimization problem that jointly identifies a small group of cells and a corresponding subset of specific genes. The proposed method extends the max-sum submatrix problem to yield genes that are, for instance, highly expressed inside a small number of cells, but have a low expression in the remaining ones.

**Results:**

We show through controlled experiments on scRNA-seq data that the MicroCellClust method achieves a high *F*_1_ score to identify rare subpopulations of artificially planted human T cells. The effectiveness of MicroCellClust is confirmed as it reveals a subpopulation of CD4 T cells with a specific phenotype from breast cancer samples, and a subpopulation linked to a specific stage in the cell cycle from breast cancer samples as well. Finally, three rare subpopulations in mouse embryonic stem cells are also identified with MicroCellClust. These results illustrate the proposed method outperforms typical alternatives at identifying small subsets of cells with highly specific expression profiles.

**Availabilityand implementation:**

The R and Scala implementation of MicroCellClust is freely available on GitHub, at https://github.com/agerniers/MicroCellClust/ The data underlying this article are available on Zenodo, at https://dx.doi.org/10.5281/zenodo.4580332.

**Supplementary information:**

Supplementary data are available at *Bioinformatics* online.

## 1 Introduction

**Fig. 1.**
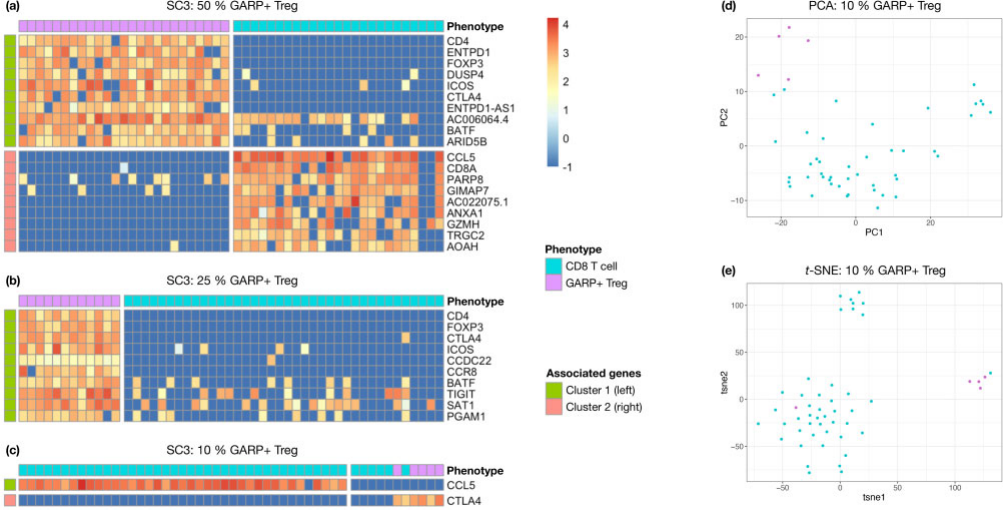
(**a–c**) SC3 correctly clusters the GARP+ Tregs (purple) separately from the CD8 T cells (turquoise) whenever their relative proportion is either 50/50% (a) or 25/75% (b). SC3 fails to identity the GARP+ Tregs as a separate and specific cluster when their relative proportion is only 10% of the cells (c). The reported genes are the marker genes identified by SC3 for each cluster. Expression values are log-normalized (${\;\text{log}\;}_{10}(x+0.1)$). (**d, e**) 2-D representation of GARP+ Tregs (purple, 10% of the data) versus CD8 T cells (turquoise, 90%) from scRNA-seq data after dimensionality reduction. Cluster separation is poor with PCA (d). A better separability is observed with *t*-SNE (e) but this method fails at identifying all GARP+ Tregs as forming a separate cluster

Next-generation single-cell sequencing technologies, such as scRNA-seq, provide an important source of data in nowadays medical research. Indeed, cell tissues present a high heterogeneity, which can be analyzed by means of single-cell data. Unsupervised clustering is a common task when analyzing scRNA-seq data. This consists in grouping cells using their expression values to highlight subpopulations in the cell tissue. Several techniques have been developed specifically toward this objective (Kiselev et al., 2019). They generally tend to group cells in relatively large clusters, but therefore tend to miss subpopulations which only amount for a small fraction of the cells.Figure 1a–c exhibits such a behavior when running SC3 (Kiselev et al., 2017), a popular method designed for single-cell clustering, on a collection of samples made of activated (GARP+) regulatory T cells and CD8 T cells from the same human patient. These two types of lymphocytes have very distinct functions, which should be reflected in their gene expression. The SC3 method has no trouble to distinguish between both cell types when their relative proportion is, by design here, 50/50. Yet, when the GARP+ Tregs only represent a smaller fraction of the data (here 10%), SC3 clearly fails to identify them as forming a separate and specific cluster.

An alternative and semi-automatic way to identify small subpopulations of cells could rely on dimensionality reduction methods to represent these cells in a 2D-space. One could visually inspect such representation to spot specific clusters. Figure 1 illustrates such an example with GARP+ Tregs (10%) and CD8 T cells (90%) after using (d) PCA (Wold et al., 1987) or (e) *t*-SNE (Maaten and Hinton, 2008), respectively a linear and a non-linear dimensionality reduction method. Cluster separation is relatively poor with PCA on this example, and it would be difficult to identify the GARP+ Treg subpopulation without any kind of supervision (the color code is only available for illustration purpose in such a controlled experiment). The *t*-SNE produces more distinct clusters here, but also fails to perfectly separate the GARP+ Tregs from CD8 T cells.

The previous examples illustrate the need for dedicated methods, beyond generic (bi)clustering methods (Xie et al., 2019), to identify small and specific cell subpopulations from scRNA-seq data. RaceID (Grün et al., 2015) and GiniClust (Jiang et al., 2016) are two popular methods to address this question. They perform a complete clustering of the data but specifically focus on the discovery of small clusters.

The original RaceID first runs *k*-means to divide the dataset into large clusters. Then, it identifies possible outliers inside each cluster from the count variability of each gene among the cells in the cluster. This variability is compared to a background model, computed over all cells, that accounts for technical and biological noise. RaceID considers a cell as an outlier if the count probability is below a predefined threshold for a certain amount of genes. The outlier cells are then grouped into small clusters based on expression correlation. RaceID3 (Herman et al., 2018) provides several adaptations to the original method to improve classification, including a univariate feature selection step before clustering.

GiniClust first performs a gene selection based on a Gini index for rare cell type identification. The value of this index is high for a gene that is differentially expressed in a small proportion of the data. GiniClust identifies ‘high Gini genes’ that are differentially expressed in a limited number of cells, and performs a clustering based on these genes. GiniClust3 (Dong and Yuan, 2020) scales up to large-scale datasets by replacing the clustering step by community detection algorithms for large networks.

Both RaceID(3) and GiniClust(3) are univariate methods in the sense that the gene expression analysis (either assessed through a count probability or a Gini index) is performed gene by gene. Such methods thus plainly ignore the possible dependence between different gene expression values when looking for rare subpopulatons of cells but rather consider each gene as playing an independent role. These methods are therefore computationally efficient but are likely to miss interesting patterns when a set of genes forms a specific expression altogether.

FiRE and scAIDE are two large-scale methods recently proposed. FiRE (Jindal et al., 2018) assigns a rareness score to each cell after selecting the 1000 most variable genes. It uses the sketching technique, which randomly projects cells to low-dimensional bit signatures and groups similar one together. Multiple sketching runs are aggregated to produce a rareness score. scAIDE (Xie et al., 2020) first learns an autoencoder to embed the genes into 256 dimensions. It then applies a random projection hashing based *k*-means algorithm on the resulting data to identify rare cell types. These two methods attempt to identify rare cells without associating them to marker genes at the same time. Instead, they rely on a univariate filter, such as the Wilcoxon’s rank-sum test, applied *a posteriori* on the spotted cells to discover marker genes.

We propose here MicroCellClust, a new method searching for relevant expression patterns in a multivariate way. More specifically, MicroCellClust looks for a relevant subset of columns and of rows in the data matrix storing the expression values for each cell and each gene, respectively. A natural multivariate objective to be optimized is the sum of expression values within the selected submatrix. This is exactly the max-sum submatrix problem which, despite its NP-hard nature, has been shown to be effective to identify gene-specific subgroups from expression data (Branders et al., 2019). As such, it is not designed for rare subpopulation identification as the maximization identifies large subgroups. MicroCellClust extends this approach by refining the objective function to be optimized and by adding useful constraints to specifically search for *rare* and *highly specific* patterns of expressions within small subpopulations of cells.

Optimizing the sum of selected entries in the data matrix naturally leads to select *highly expressed* genes in the selected cells, whenever the data matrix represent the original scRNA-seq count values (or a log-normalized version of them). Considering the *opposite* of these original values would lead to select cells with *low* expressed genes after solving the same problem. Other data normalizations can also lead to other interesting patterns (e.g. the genes departing the most of the median expression values for each cell). Nevertheless, we stick here for clarity to the original interpretation with selected entries corresponding to jointly *high* expression values. This choice is also consistent with the nature of scRNA-seq data because of the dropout phenomenon (Ziegenhain et al., 2017) which could lead to many false positives whenever one looks for low expressed values.

## 2 The MicroCellClust method

A scRNA-seq dataset can be represented as an expression matrix $M\in R^{\left|G|\times |C\right|}$, with G the set of rows associated to the genes and C the set of columns associated to the cells. The *m_ij_* entry of this matrix is here assumed positive whenever the gene *i* is expressed in the cell *j*, and negative otherwise. This requires to choose a threshold on the raw expression values (i.e. normalized read counts) to specify a minimal expression level above which a gene is considered expressed, and to scale the data accordingly. One typically considers a log-scaling ${\;\text{log}\;}_{10}(x+0.1)$, with *x* the raw expression value and 0.9 as expression threshold in this example. Negative values of such log-normalized data represents genes that are negligibly expressed, or not expressed at all.

The goal is to select a subset of genes I⊆G and a corresponding subset of cells J⊆C, in other words, a bicluster (*I*, *J*), representative of a small subpopulation of, by default, highly expressed cells and highly specific genes. This goal is formalized below as a constrained optimization problem for which an optimal solution is denoted by $\left(I^{*},J^{*}\right)$:

(1) $$\left(I^{*},J^{*})=\underset{I\subseteq GJ\subseteq C}{\text{argmax}}\sum_{i\in I}(\sum_{j\in J}m_{ij}-\kappa \sum_{k\in C\backslash J}\max\{0,m_{ik}\}\right)$$

 

(2) $$\text{such}\;\text{that}\;\;\frac{\left|\{(i,j)\;|\;i\in I,j\in J,m_{\text{ij}}<0\}\right|}{\left|I|\cdot |J\right|}\leq \mu$$

The objective function in Equation (1) is composed of two terms. Maximizing the first term, i.e. $\sum_{i\in I}\sum_{j\in J}m_{ij}$ corresponds to the max-sum submatrix problem (Branders *et al.*, 2019). One searches for a bicluster for which the sum of all the corresponding expression values is maximal. The global sum criterion allows for variations within the expression values, which is well suited to scRNA-seq data since it is typically subject to technical and biological noise (Todorov and Saeys, 2019). An unusually high expression of a gene in a particular cell, e.g. due to transcriptional bursting, would have little influence on the bicluster found since expression values are not compared in a pairwise fashion. Moreover, the sum criterion allows for some genes within the selected bicluster to be lowly, or even negatively, expressed for some cells as long as their inclusion increases the objective value globally, which is consistent with the dropout phenomenon. In other words, the sum allows for exceptions within the selected bicluster as only the global influence of the selected cells and selected genes matters. Single-cell data is however also often sparse in terms of expressed values. The parameter *μ* (typically fixed to 10%), included in the additional constraint (2), controls the proportion of negative values allowed in the solution.

As such, the max-sum objective does not guarantee the solution to be highly specific. In other words, the genes selected within the bicluster could also be highly expressed in other cells. The second term prevents such situation by penalizing negatively the out-of-cluster cells (k∈C\J) that would express the same genes positively (hence the max{0,.} operation). The parameter *κ* controls the relative influence of these two terms within the objective function. The higher *κ* the fewer genes will tend to be included in the solution. Our results described in Section 3 suggest that selecting 10–30 genes is often relevant from a biological viewpoint. We suggest $\kappa =\frac{100}{\left|C\right|}$ and μ=10% as default values. Our Supplementary Material includes a detailed analysis of the influence of these parameters.

**Fig. 2.**
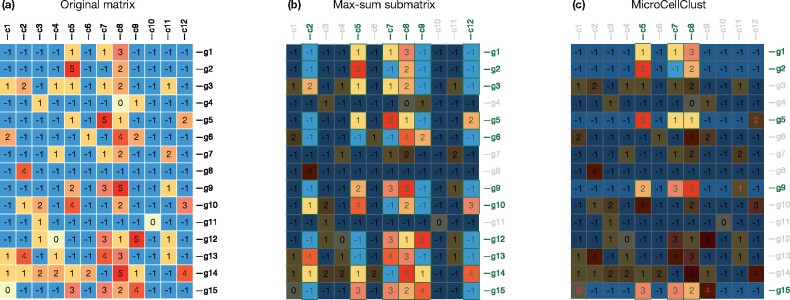
(**a**) A toy example of scRNA-seq data after log-normalization and rounding to integer values for clarity of this example. (**b**) The optimal solution to the (unconstrained) max-sum submatrix problem. (**c**) The MicroCellClust solution with *κ *= 1 and μ=10%

Figure 2a represents a toy example of scRNA-seq data made of 12 cells and 15 genes. The unconstrained max-sum submatrix solution (Fig. 2b) forms a relatively large bicluster with a low specificity. It includes, for instance, the cells *c*_2_ and *c*_12_ as they positively contribute to the objective (the sum of the selected entries in their respective column is positive) despite many negative values and a low similarity with the other selected cells. Figure 2c represents the MicroCellClust solution, with *κ *= 1 in the objective function (1) under the constraint (2) with μ=10%. The three cells inside the selected bicluster have a similar expression of their five selected genes. These five genes are also specifically expressed in this bicluster with only a few out-of-cluster expressions (red digits in one out-of cluster cell, respectively for *g*_5_, *g*_9_ and *g*_15_). Other genes, such as *g*_3_, are no longer selected since their out-of-cluster positive expression implies a negative contribution to the objective function.

Problem (1), constrained according to (2), requires the expression matrix to be sparse. Indeed, many genes could be expressed in nearly all cells. Such a situation would lead to an optimal solution being a large bicluster that would no longer be specific to a small population of cells. In the limit, all cells could be part of the selected bicluster (J=C) and the second term of (1) would vanish. MicroCellClust therefore filters out initially any gene expressed in more than *x*% of the cells (typically 25%, which generally keeps around 80% of the genes). This initial filtering is motivated by the search of gene markers of specific subpopulations rather than generic markers of high expression throughout the cell population.

We propose a constraint programming solver implemented in R and Scala to find an optimal solution to problem (1), (2). It is inspired from the CPGC algorithm (Branders et al., 2018) used to solve the max-sum submatrix problem. In particular, the notion of implicit search space also applies to our constrained [see (2)] and extended objective [see (1)]. A naive implementation would indeed search explicitly all possible subsets of columns (cells) and of rows (genes) and evaluate $2^{\left|G|+|C\right|}$ potential biclusters. It turns out that whenever one dimension is fixed (typically the cells), the optimal solution along the other dimension (the genes) can be found in linear time. Consequently, the actual search space size to consider is $O(|G|\cdot 2^{\left|C\right|})$.

Heuristics are used to evaluate at an early stage of the search which combinations of cells are most likely to produce an optimal solution. Specifically, the solver follows a breadth-first search strategy: evaluate all possible biclusters of two cells, next biclusters of three cells, *etc*. At each level, the solver selects the biclusters with highest objective values to continue the search. Only supersets of the set of cells they include are evaluated at the next level. This strategy is well suited to problem (1), (2). Due to the high specificity of the optimal bicluster, any subset of its cells is expected to form a promising solution the objective value of which is above average. Empirical observations illustrate that the distribution of objective values, at a given level of the search, roughly follows a power law, which suggests to ignore the long tail of the distribution.

Comparative results between this strategy and an exhaustive search show that such a heuristic search produces the same results with an $O(|C{|}^{2})$ complexity. This substantially shortens the computing time: less than 2 seconds for a dataset of 50 cells × 10 000 genes; around 10 min for 1000 cells (see details in Supplementary Material). These results have been produced on MacBook Pro laptop (Mac OS 10.15.7; 2.7 GHz Intel Core i7 CPU; 16 GB RAM).

## 3 Results

We illustrate below the benefits of using MicroCellClust to identify small and highly specific subpopulations of cells and their associated marker genes from scRNA-seq data. The first results allow one to compare MicroCellClust with the RaceID3, GiniClust and scAIDE methods. Such a comparison is conducted in a controlled setting to spot few GARP+ Tregs cells among CD8 T cells, as in our motivating example described in Section 1. GiniClust3 and FiRE are not effective to solve this task as commented below. A second controlled experiment aims at spotting rare Jurkat cells among 293T cells from human cell lines. In this case, the fraction of rare cells artificially planted varies from .5% to 10%. The experiments are conducted on datasets with up to 1000 cells which allows further comparisons with methods specifically designed to deal with large-scale data such as FiRE, GiniClust3 and scAIDE. Next, we report how MicroCellClust highlights a small subpopulation with a specific phenotype inside CD4 T cells from breast tumor samples, as well as a small subpopulation linked to cell cycle activity among activated Tregs. Finally, we report how MicroCellClust identifies several relevant subpopulations in mouse embryonic stem cells.

### 3.1 Identification of rare GARP+ Tregs among CD8 T cells

The original data is composed of T lymphocytes extracted from a human breast tumor, sorted by flow cytometry into CD8 T cells and activated regulatory T cells and processed following the SmartSeq2 protocol (Picelli et al., 2014) (see details in Supplementary Material). Raw expression values are log-transformed (${\;\text{log}\;}_{10}(x+0.1)$), except for RaceID3 and Giniclust which require raw expression data. We design a controlled dataset by sampling uniformly at random five GARP+ Tregs (from the 96 available ones) and 45 CD8 T cells (from the 83 available ones after quality control). Such a random sampling is repeated independently 100 times and, for each run, the scRNA-seq data matrix contains 50 cells and ≈18 000 genes on average (MicroCellClust filters out ≈2500 genes expressed in more than 25% of the cells). We assess to which extent each method is able to identify as a separate and specific cluster the small population of GARP+ Tregs (10% of the cells) among the CD8 T cells. These two kinds of lymphocytes have very distinct functions. Yet, clustering them from their expression values into separate and specific subpopulations is not necessarily straightforward, as illustrated in Figure 1.

RaceID3 and GiniClust perform a full clustering of the data, for which each cell is assigned to one cluster. In contrast, MicroCellClust spots a specific subpopulation of cells leaving the other cells not clustered. Yet, it is straightforward to run MicroCellClust again on the remaining cells. Repeating this strategy *k* times defines the top-*k* candidate subpopulations identified by MicroCellClust (it turns out that considering the top-2 biclusters is sufficient to find a Treg related cluster for every run, as illustrated below). For all tested methods, the cluster with the highest proportion of GARP+ Tregs is considered to be the solution found for a specific run.

Table 1 reports the precision (=the proportion of cells included in the solution which are actual Tregs) and recall (=the proportion of the five Tregs to be found actually included in the solution) for each method. Both metrics and their harmonic average, or *F*_1_ score, are averaged over the 100 independent runs. The first column (Nb. ⊆) indicates the number of runs for which the solution is composed exclusively of Tregs (100% precision). The second (Nb. =) column indicates the number of runs for which all five Tregs exactly form the solution (100% precision and recall). MicroCellClust clearly outperforms the competing methods with a *F*_1_ score equal to 76%, and even equal to 92% when considering the top-2 strategy. MicroCellClust top-2 also identifies perfectly the 5 GARP+ Tregs in 54 out of the 100 runs. This is much less the case with RaceID3 and GiniClust which illustrates that these methods are far less efficient at finding rare and specific subpopulations.

**Table 1.** Identification results of rare GARP+ Tregs among CD8 T cells, averaged over 100 independent runs

Method	Nb. ⊆	Nb. =	Precision	Recall	*F*_1_ score
MicroCellClust top 1	71	45	0.79	0.74	0.76
MicroCellClust top 2	**86**	**54**	**0.96**	**0.89**	**0.92**
RaceID3	48	2	0.75	0.47	0.55
GiniClust	9	0	0.35	0.59	0.37
scAIDE	1	0	0.20	0.95	0.32

(Nb. ⊆) reports the number of runs with 100% precision.

(Nb. =) reports the number of runs with 100% precision and recall.

Bold results indicate which method outperforms the others for each metric.

**Fig. 3.**
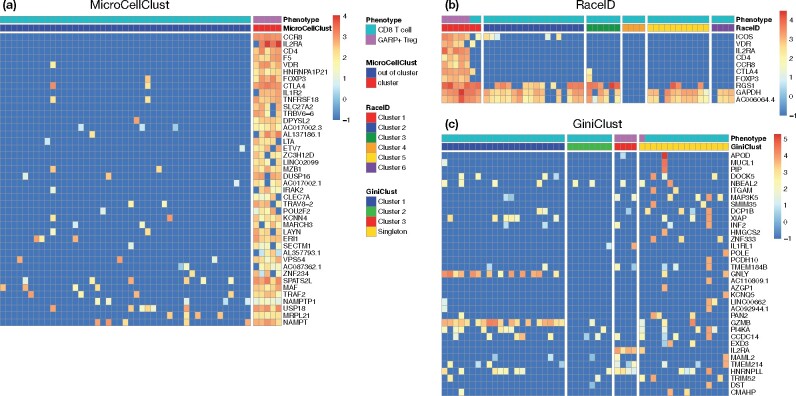
Representative results of GARP+ Tregs identification within CD8 T cells. (**a**) Marker genes identified by MicroCellClust in a typical run where it identifies the five GARP+ Tregs perfectly. (**b**) Marker genes for cluster 1 found by RaceID3 in a typical run. RaceID3 correctly identified Treg-associated genes, but also includes CD8 T cells in the same cluster. The last 3 genes are also highly expressed in other cells. (**c**) High Gini genes identified by GiniClust for a representative run. Four out of five Tregs are correctly identified in a separate cluster but the marker genes are not particularly expressed in this cluster

Figure 3a reports the bicluster found by MicroCellClust for a representative run. The five Tregs are perfectly identified and the selected subset of marker genes is informative. For instance, the marker genes include IL2RA and FOXP3, which are clear indicators of Treg cells and CCR8, which is specific to Tregs present in the breast (Plitas et al., 2016).

Such a result is very rarely obtained with RaceID3. In most cases, the Tregs are split into different clusters and/or end up being clustered with CD8 T cells. The outlier detection procedure behaves poorly here as RaceID3 often identifies only a subset of Tregs as outliers, resulting in mixed clusters. Figure 3b illustrates a good performance run for RaceID3 that correctly identifies Treg related genes, such as VDR, IL2RA, CD4, CCR8 and FOXP3, also present in the MicroCellClust solution. However, two CD8 T cells are also present in the solution found and the last three marker genes are also highly expressed in many cells from the other clusters.

GiniClust performs poorly in this controlled experiment. GiniClust [with the *P*-value cutoff of the Gini index tuned to 0.005 since the default cutoff ($10^{-4}$) leads to select very few genes, none at all for some run] identifies ≈35 high Gini genes on average over 100 runs. Yet, it always fails at identifying exactly the five GARP+ Tregs. Figure 3c illustrates a best run, in relative terms, for GiniClust. For this run, four out of five Tregs are correctly identified in a separate cluster but the marker genes are not particularly expressed in this cluster. In other words, GiniClust fails at identifying highly specific genes which would be together informative of the identified cell clusters. This is most likely due to the univariate gene selection performed in this method which contrasts drastically with the MicroCellClust approach.

The methods for large-scale data are performing very poorly in this experiment. scAIDE has a very low precision with the Tregs assigned to large clusters of 25 cells on average. FiRE returns the same score to each cell and is therefore useless for this data. GiniClust3 finds only 1 candidate marker gene, no matter which Gini cut-off is considered, and is therefore useless as well.

### 3.2 Identification of rare Jurkat cells among 293T cells

We further asses the performance of MicroCellClust while varying data sizes (from 50 to 1000 cells) and rare cell proportions using a dataset containing two human immortalized cell lines: 293T and Jurkat cells (Zheng et al., 2017; Zheng et al., 2017). These two cell types were mixed in vitro and a single-nucleotide variant has been used to determine the lineage of each cell, resulting in a total of 1540 293T cells and 1694 Jurkat cells. We design a controlled experiment by sampling at random rare populations of Jurkat cells (from 0.5% to 10% of the data, with a minimum of 5 cells) and abundant populations (up to 1000 cells) of 293T cells and ≈15 000 genes. MicroCellClust uses ≈9500 genes expressed in at most 25% of the cells. For each combination, 10 independent runs are performed. For this experiment, the *κ* parameter of MicroCellClust has been chosen as $\kappa =\frac{100}{\left|C\right|}$ which produces results with ≈20 marker genes, regardless of the size of the number of rare cells in the identified bicluster.

Table 2 reports the *F*_1_ score of each method averaged over 10 runs. MicroCellClust achieves a high *F*_1_ score (≥90%) in each case (the top-1 solution always produces a Jurkat related bicluster during this experiment). This is not the case for the other methods. The methods designed for large-scale datasets perform poorly whenever the total number of cells is less or equal to 200. For larger datasets, with at least 500 cells, they may perform well but their performance significantly drops when the subpopulation to identify only contain a few cells (5–10). These results illustrate that the performance of MicroCellClust is stable with respect to the total number of cells. It is clearly the best performing method to identify a particularly rare subpopulation of cells.

**Table 2.** Identification results of rare Jurkat cells among 293T cells: average *F*_1_ score over 10 independent runs

Total size	Rare cells		MCC	RaceID3	FiRE	GiniClust3	scAIDE
50	5	(10%)	**0.99**	0.71	0.00	0.00	0.74
100	10	(10%)	**0.99**	0.68	0.00	0.00	0.47
100	5	(5%)	**0.97**	0.72	0.16	0.00	0.18
200	20	(10%)	**0.96**	0.67	0.13	0.00	0.57
200	10	(5%)	**0.95**	0.59	0.41	0.00	0.16
200	5	(2.5%)	**0.95**	0.66	0.54	0.00	0.03
500	50	(10%)	0.93	0.46	0.78	**0.99**	**0.99**
500	25	(5%)	0.95	0.45	0.80	0.88	**0.99**
500	10	(2%)	**0.95**	0.51	0.56	0.01	0.83
500	5	(1%)	**0.92**	0.60	0.41	0.71	0.06
1000	100	(10%)	0.91	0.41	0.92	**1.00**	0.72
1000	50	(5%)	0.91	0.43	0.77	**1.00**	0.85
1000	20	(2%)	0.95	0.49	0.51	**0.99**	0.98
1000	10	(1%)	**0.92**	0.53	0.33	0.50	0.30
1000	5	(0.5%)	**0.93**	0.51	0.19	0.62	0.05

*Note*: MCC stands for MicroCellClust.

Bold results indicate which method outperforms the others for each experiment.

### 3.3 Identification of a T regulatory related subpopulation within CD4 T cells

We further illustrate the potential of MicroCellClust to highlight a small subpopulation among cells of interest, by analyzing a set of 182 CD4 T cells from the breast sample under study in Section 3.1. It contains 93 tumor and 89 healthy cells (after quality control) and 16 737 genes expressed in at most 25% of the cells. The objective is to check whether any subpopulation with a specific function can be identified among these CD4 T cells, and to link it to the immune response against breast cancer. The healthy cells can serve here as a control to see whether the identified subpopulation only contains tumor cells, i.e. with a function specific to a tumor.

**Fig. 4.**
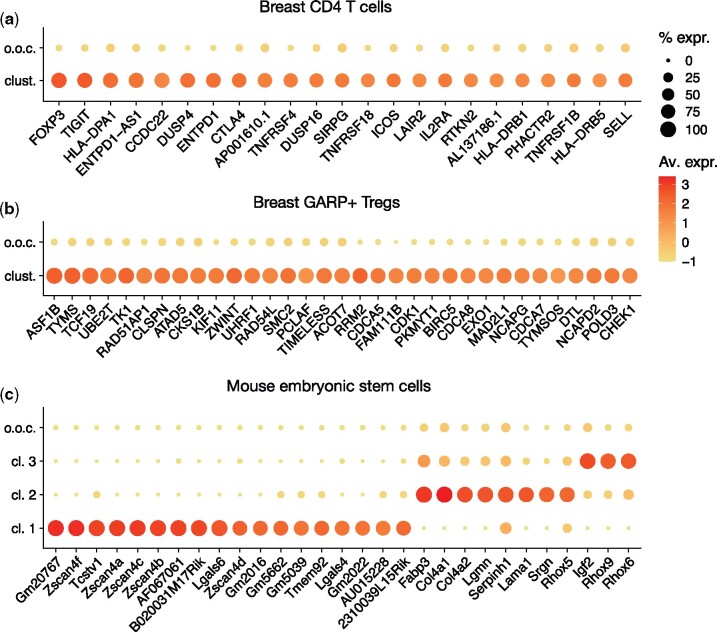
MicroCellClust identifies subpopulations in human breast cancer cells, namely (**a**) a subpopulation within CD4 T cells with marker genes related to GARP–Tregs, and (**b**) a subpopulation withing GARP+ Tregs with marker genes related to cell division. (**c**) MicroCellClust identifies three subpopulations in mouse embryonic stem cells. In the above plots, ‘o.o.c’ refers to out-of-cluster expression values and the other rows represent the identified biclusters. Full heatmaps are included in Supplementary Material

MicroCellClust identifies a cluster of 23 cells originating from the tumor, characterized by the expression of Treg associated genes such as FOXP3 and IL2RA (Fig. 4a). Interestingly, these cells were not annotated as GARP+ Tregs by the flow cytometry sorting since they did not express GARP. However, their gene expression clearly indicate Treg-related functions. This is confirmed by a Gene Ontology enrichment analysis (Ashburner et al., 2000; The Gene Ontology Consortium, 2019) (see detailed results in Supplementary Material). We conjecture that GARP is transiently expressed after Treg activation, and that the identified cells might be related to the GARP+ Tregs by being resting or not recently activated Tregs. Indeed, five cells from the identified cluster appear to have the same T cell receptor (TCR) sequences than GARP+ Tregs, which supports our hypothesis. To sum up, MicroCellClust enables us to identify a set of resting or not recently activated Tregs among CD4 T cells, a subpopulation that had not been sorted out by flow cytometry.

MicroCellClust returns a bicluster with five marker genes when using $\kappa =0.5\approx \frac{100}{\left|C\right|}$ and μ=10%, including FOXP3 which already suggests a Treg related function. This relatively low number of marker genes suggests to decrease *κ* to 0.3, providing a solution with 12 cells and 18 genes, including IL2RA which further supports the Treg interpretation. Some cells express a significant proportion of these marker genes but are not included in this bicluster. Such a behavior can probably be explained by the presence of some dropouts in the scRNA-seq data, and the transient nature of Treg activation. This suggests to increase the *μ* parameter to 30%. The identified subpopulation is *a posteriori* validated by the fact that the bicluster found includes five cells sharing a TCR sequence with a GARP+ Treg (see further details in Supplementary Material).

### 3.4 Identification of a cell-cycle related subpopulation in tumor GARP+ Tregs

Our next example illustrates the use of MicroCellClust to search for a potential subpopulation inside a set of GARP+ Tregs among breast tumor samples from two different patients. The scRNA-seq data represents here 168 cells and 13 772 genes expressed in at most 25% of the cells.

MicroCellClust with values $\kappa =0.6\approx \frac{100}{\left|C\right|}$ and *μ *= 10% identifies a subpopulation of 21 cells which forms a specific cluster associated to numerous cell division related genes (Fig. 4b). A GO enrichment analysis indeed highlights cell division functions (see Supplementary Material). Such an interpretation makes sense since GARP+ Tregs represent a population of recently activated Tregs, and T cell activation usually leads to cell proliferation. MicroCellClust thus reveals the existence of dividing Tregs inside breast tumor tissues, an observation definitely worth further investigation.

On this data, the resulting bicluster is stable with respect to the *κ* parameter. When increasing *κ*, the exact same cell subpopulation is identified, while the number of genes decreases (33 for κ=0.6 down to 26 for *κ *= 1) since out-of-cluster expression is more penalized with a higher *κ* (see further details in Supplementary Material).

### 3.5 Identification of rare subpopulations in mouse embryonic stem cells

Our last case study shows the result of MicroCellClust on a dataset of mouse embryonic stem cells 4 days after leukemia inhibitory factor withdrawal (Klein et al., 2015; Klein et al., 2015). It contains 683 cells and 19 749 genes expressed in at most 25% of the cells. The top-*k* strategy, with κ=0.1 and μ=10%, is used to identify three distinct subpopulations (Fig. 4c). The first bicluster is composed of 5 cells and 18 marker genes, including genes from the Zscan4 family and Tcstv1, which have been associated to 2 C-like cells (Macfarlan et al., 2012). To evaluate the reproducibility of this very small subpopulation (<1% of the cells) when resampling data, 500 cells are drawn at random while forcing the five identified cells to be included. This subpopulation is correctly identified again over 10 independent runs. Moreover, it is stable with respect to the choice of *κ* value, as the same bicluster is identified for *κ* between 0.08 and 0.2.

The top-*k* strategy returns two additional subpopulations. The first one contains 14 cells and 8 marker genes, among which primitive endoderm markers Col4a1, Col4a2 and Lama1. Finally, a subpopulation of 61 cells with three highly expressed marker genes is identified, including maternally imprinted genes Rhox6 and Rhox9. The 3 subpopulations identified by MicroCellClust on this dataset have been identified in the original publication using additional *prior* knowledge. This confirms the ability of MicroCellClust to identify subpopulations with biologically relevant gene combinations.

## 4 Conclusion

We propose MicroCellClust, a new data mining method to identify *small* subpopulations of cells with *specific* gene expression. MicroCellClust is a multivariate method that jointly looks for a small group of cells and the corresponding marker genes. MicroCellClust solves a combinatorial optimization problem specifically tailored to this problem. We report several experiments illustrating the benefits of MicroCellClust to identify small and highly specific biclusters from scRNA-seq data. The reported experiments clearly show the higher precision and recall with MicroCellClust as compared to existing methods, especially when the rare subpopulation contains only a few cells. Moreover, MicroCellClust highlights several subpopulations of interest in T cells extracted from breast tumor samples, as well as in mouse embryonic stem cells. The relevance of these subpopulations is discussed and biologically motivated in each case.

The proposed optimization problem relies on two parameters: *κ* and *μ*. The reported experiments suggest to choose $\kappa =\frac{100}{\left|C\right|}$ and μ=10% by default. These parameters may be further tuned in order to refine the solution, typically based on the number of returned marker genes. Opting for ≈20 such genes is good rule of thumb in this regard. In all our experiments, such a tuning has been easily conducted without the need of any additional prior knowledge.

MicroCellClust has been assessed so far on scRNA-seq datasets including thousands of cells and tens of thousands candidate marker genes. This is motivated by the search for rare subpopulations in data that is often limited in availability (e.g. intra-tumoral Tregs). The $O(|C{|}^{2})$ complexity of MicroCellClust easily scales to such data, but would become less practical for a significantly larger number cells. Scaling up MicroCellClust is part of our future research.

The method introduced here is motivated by the analysis of scRNA-seq data, which is our primary objective in this work. Yet, from a computational viewpoint, MicroCellClust could be seen as a generic biclustering approach for finding *rare patterns* that could be applied to alternative data sources.

MicroCellClust takes as input a single data matrix representing all gene expression values for a population of cells typically coming from a single biological sample. Using the same procedure one can easily analyze the data coming from a few samples simply put together. A natural extension would consider the sample (or patient) identity as a third dimension, beyond genes and cells. It would indeed be interesting to find specific subpopulations of cells and their associated marker genes that would also be common across different conditions or patients. Our future research work will adapt the optimization problem introduced here to address this generalized objective.

## Supplementary Material

btab239_Supplementary_Data
