# Supplementary material for: MicroCellClust: mining rare and highly specific subpopulations from single-cell expression data
Source: Bioinformatics. 2021 Apr 8;37(19):3220–7. doi: 10.1093/bioinformatics/btab239 (PMC8504615; doi:10.1093/bioinformatics/btab239)
Supplement: btab239_Supplementary_Data [file btab239_supplementary_data.pdf]

MicroCellClust: mining rare and highly specific subpopulations from single-cell expression data

Supplementary material

A. Gerniers, O. Bricard and P. Dupont

Contents

A Breast cancer data description . . . 1
B Additional figures for the case studies . . . 2
B.1 Treg related subpopulation in CD4 T cells (section 3.3 of the main manuscript) . . . 2
B.2 Cell-cycle subpopulation in tumor GARP+ Tregs (section 3.4 of the main manuscript) . . 2
B.3 Mouse embryonic stem cells (section 3.5 of the main manuscript) . . . 2
C Gene Ontology enrichment analysis . . . 7
C.1 GO functions for the Treg related subpopulation within CD4 T cells . . . 7
C.2 GO functions for the cell-cycle subpopulation in GARP+ Tregs . . . 7
D MicroCellClust implementation . . . 8
D.1 Constraints . . . 8
D.2 Heuristic search evaluation . . . 8
D.3 Values of the heuristic parameter t . . . 10
D.4 Execution time and memory analysis . . . 10
E Additional results . . . 10
E.1 Geneset reproducibility for Treg/CD8 experiment . . . 10
E.2 Max-sum submatrix result . . . 10
E.3 Illustration of top-2 result in Treg/CD8 experiment . . . 15
E.4 Identification of CD4 subpopulation among tumor cells only . . . 15

A Breast cancer data description

The data used in sections 3.1, 3.3 and 3.4 of the main manuscript is composed of T lymphocytes extracted from a human breast tumor and a healthy breast tissue. These cells are sorted in a 384-well plate by flow cytometry according to three distinct phenotypes: CD8 T cells (CD3+ CD8+), CD4 T cells (CD3+ CD4+), and activated regulatory T cells (CD3+ CD4+ CD25+ GARP+). A total of 96 cells from each phenotype and tissue origin combination are processed following the SmartSeq2 protocol [1] to generate scRNA-seq data on the Illumina HiSeq4000 platform. 72 GARP+ tumorous cells of another patient are processed in the same way. A summary is given in table 1. The `cleanhop` algorithm [2] is used to remove potential misassigned reads due to index hopping. It subtracts to the number of reads, for each cell and for each gene, a percentage (0.5% by default) of the sum of the reads associated with the gene among all the cells sharing the same i7 index (column), and the sum of all the reads associated with the gene among all the cells sharing the same i5 index (row). Finally, read counts are normalized by the median total read count per cell.

<table>
<tr><th>Patient</th><th>Tissue_origin</th><th>FACS_phenotype</th><th>Nb. cells</th><th>3.1</th><th>3.3</th><th>3.4</th></tr>
<tr><td rowspan="4">KOBE</td><td>ExtraTumor</td><td rowspan="2">CD3+_CD4+</td><td>89</td><td></td><td rowspan="2" style="background:gray"></td><td></td></tr>
<tr><td rowspan="4">Tumor</td><td>93</td><td></td><td></td></tr>
<tr><td>CD3+_CD8+</td><td>83</td><td rowspan="2" style="background:gray"></td><td></td><td></td></tr>
<tr><td rowspan="2">CD3+_CD4+_CD25+_GARP+</td><td>96</td><td></td><td rowspan="2" style="background:gray"></td></tr>
<tr><td>JORD</td><td>72</td><td></td><td></td></tr>
</table>

Table 1: Annotations of the breast cancer data. The 4th column indicates the number of cells that where processed (after quality control) for each data subset; the 3 last columns indicate in which experiments they are used (number of the section in the main manuscript).

B Additional figures for the case studies

We present three case studies to illustrate the results of MicroCellClust on scRNA-seq data in the main manuscript, where the biological interpretation of each identified subpopulation is discussed (sections 3.3, 3.4 and 3.5). Here, we report heatmaps (figures 1, 3, 5 and 7) representing each identified subpopulation of cells with the expression of their marker genes. These figures also illustrate the influence of changing parameters κ and μ, together with graphs reporting the evolution of the number of cells and genes when changing parameter values (figures 2, 4 and 6).

B.1 Treg related subpopulation in CD4 T cells (section 3.3 of the main manuscript)

Figure 1 (a) illustrates the result when using $\kappa = 0.5 \approx \frac{100}{|\mathcal{C}|}$ and $\mu = 10\%$ on the breast CD4 T cell dataset. The presence of FOXP3 as a marker gene indicates a Treg related function. The relatively low number of 5 marker genes suggests to decrease κ to 0.3 (figure 1 (b)) in order to decrease the out-of-cluster penalty, allowing more genes to be included in the solution. Decreasing κ also tends to select fewer cells, as can be seen in figure 2. Only 12 of the 21 previously identified cells are selected with $\kappa = 0.3$ and $\mu = 10\%$. A few cells express a significant proportion of the 18 marker genes returned here but are not included in the bicluster found. This motivates to relax the non-negativity constraint and to set $\mu = 30\%$. Figure 2 also shows that with $\mu = 30\%$, the cell subpopulation is the most stable with respect to κ, as the same 24 cells are selected for $\kappa \in [0.3, 0.7]$. The identified subpopulation (figure 1 (c)) is *a posteriori* validated by the fact that the bicluster found includes 5 cells sharing a TCR sequence with a GARP+ Treg.

B.2 Cell-cycle subpopulation in tumor GARP+ Tregs (section 3.4 of the main manuscript)

Figure 3 illustrates the result with $\kappa = 0.6$ and $\mu = 10\%$ on the tumor GARP+ Treg dataset. 21 cells are identified together with 33 marker genes. This subpopulation is stable when increasing κ, as the same 21 cells are selected, but with fewer marker genes as increasing κ (check left of figure 3) results in only keeping the most differentially expressed ones.

As the results in figure 3 correspond to our suggestion of selecting 10 to 30 genes, the value $\kappa = 0.6$ seems appropriate. With a lower value (*e.g.* $\kappa = 0.5$), fewer cells would be selected (see figure 4) while several out-of-cluster cells would express a lot of marker genes in such a solution. This further confirms that the choice of $\kappa = 0.6 \approx \frac{100}{|\mathcal{C}|}$ is suitable. Increasing μ to *e.g.* 20% is not appropriate here, since most κ values produce a bicluster containing also 21 cells (see figure 2). In such cases, one tends to prefer the most restrictive μ value (*i.e.* 10%).

B.3 Mouse embryonic stem cells (section 3.5 of the main manuscript)

Figure 5 illustrates the 3 subpopulations identified by MicroCellClust in the mouse stem cell data with $\kappa = 0.1 \approx \frac{100}{|\mathcal{C}|}$ and $\mu = 10\%$. The expression of the marker genes of cluster 1 is highly differentiated. It is therefore unsurprising that the same 5 cells are selected for a large set of κ values around 0.1 (see figure 6). When relaxing μ to 20%, an extra cell, which expresses about half of the marker genes, is added (all marker genes of the $\mu = 10\%$ solution are marker genes for the $\mu = 20\%$ result; see figure 7). In particular, it expresses 4 genes of the Zscan4 family, so this extra cell could also be of interest even though is does not express a large part of the marker genes.

The number of cells and genes assigned to cluster 2 is more sensitive to variations of κ, which probably comes from the fact that the marker genes are more expressed in out-of-cluster cells. In particular, when κ gets too high, *e.g.* $\kappa = 0.12$, the penalty is too strict and only 2 genes are selected. Such a value is thus not adapted for this particular subpopulation. The result with $\mu = 20\%$ is slightly less interesting but reported in figure7 for completeness: a larger number of cells is selected but to some relevant marker genes (such as Col4a2) no longer belong to the solution.

MicroCellClust identifies a third bicluster made of 3 marker genes (Igf2, Rhox9 and Rhox6) that are expressed in a relatively significant number of cells (9% of the data). These 3 genes are also identified with different combinations of parameter values (in a varying number of cells depending on these values). Interestingly, one particular combination, $\kappa = 0.05$ and $\mu = 20\%$ identifies these genes together with 13 other genes, expressed in 21 of the 62 previously identified cells. These cells could be seen as a subcluster within the 62 cells, as they coexpress other marker genes besides Igf2, Rhox9 and Rhox6 (see cluster 3 in figure 7).

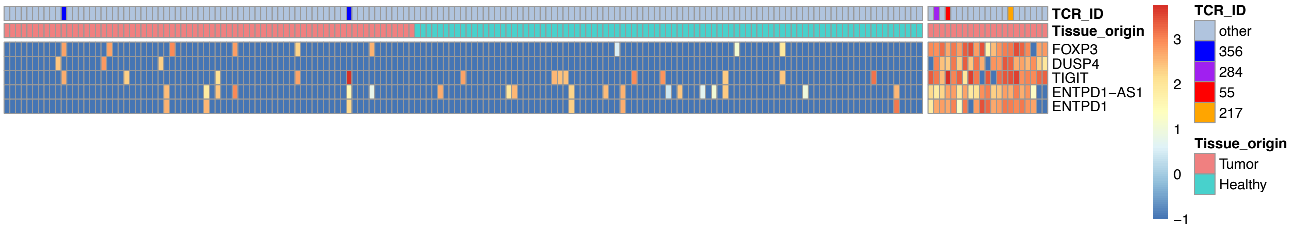

(a) $\kappa = 0.5$, $\mu = 10\%$

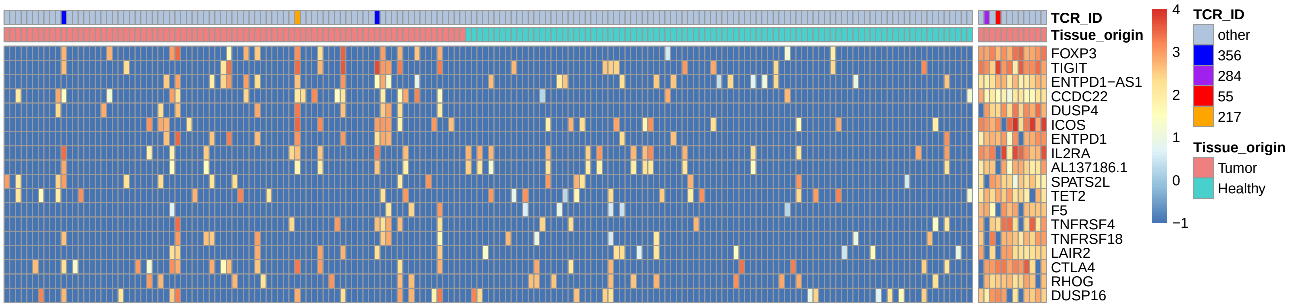

(b) $\kappa = 0.3$, $\mu = 10\%$

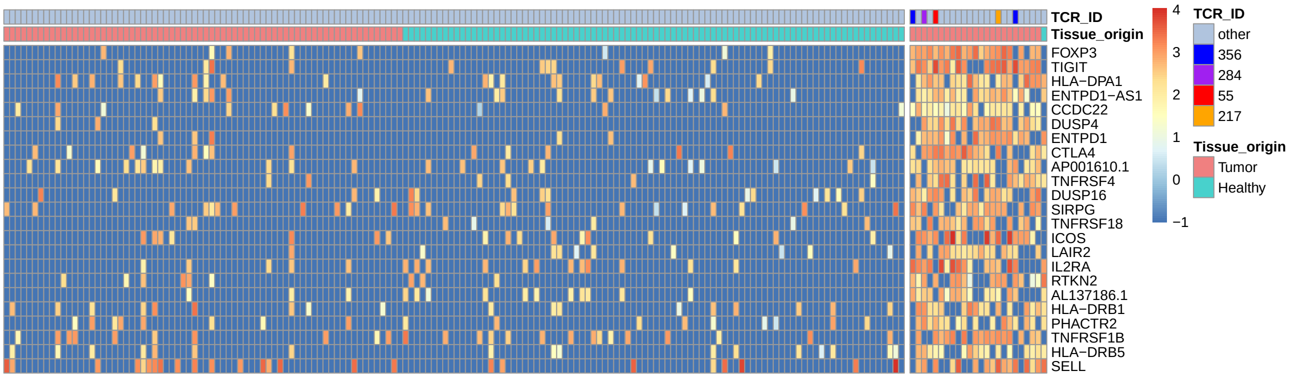

(c) $\kappa = 0.3$, $\mu = 30\%$

Figure 1: Treg related subpopulations (cells on the right) identified among CD4 lymphocytes, obtained when changing parameters κ and μ. The marker genes identified by MicroCellClust indicate that these cell-clusters are related to GARP− Tregs. Cells sharing a TCR sequence with a GARP+ Treg are reported (see first row on top of each heatmap) further confirming this hypothesis.

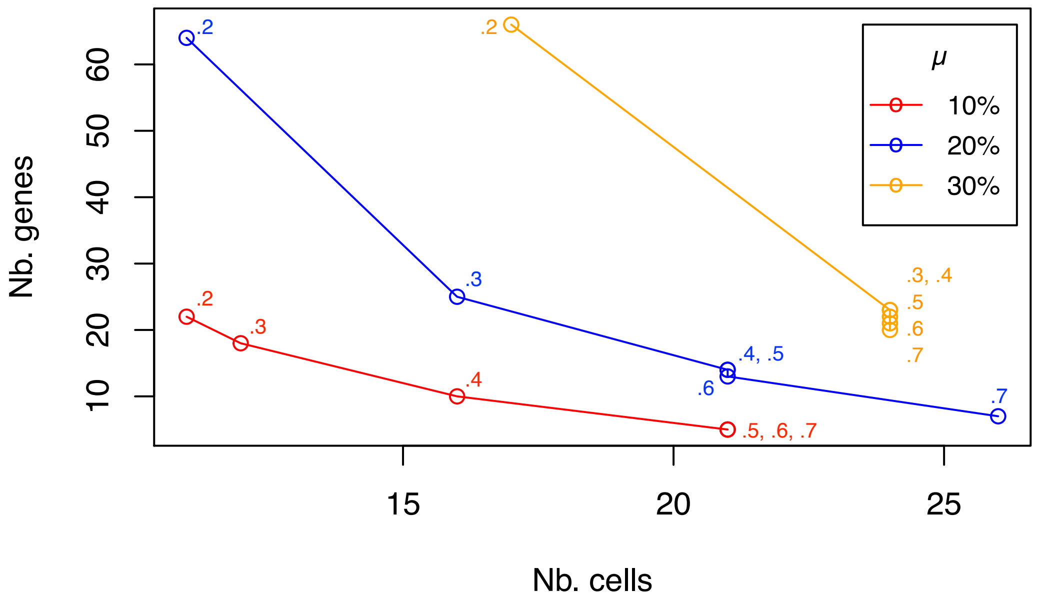

Figure 2: Number of cells and genes identified by MicroCellClust in the CD4 T cell data in function of the κ parameter (values indicated next to the dots), for three different values of the μ parameter.

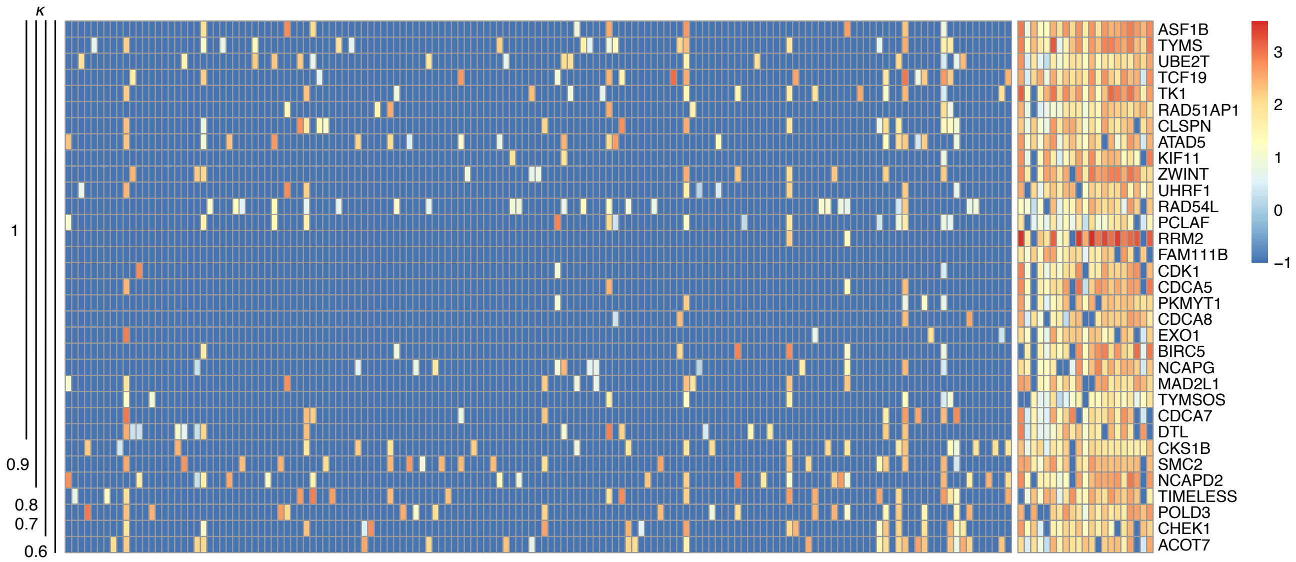

Figure 3: Cell-cycle related subpopulation among GARP+ Tregs (21 cells on the right). The identified marker genes are mostly related to cell division. Vertical bars on the left of the heatmap indicate which marker genes are selected when changing the value of κ from 0.6 to 1.

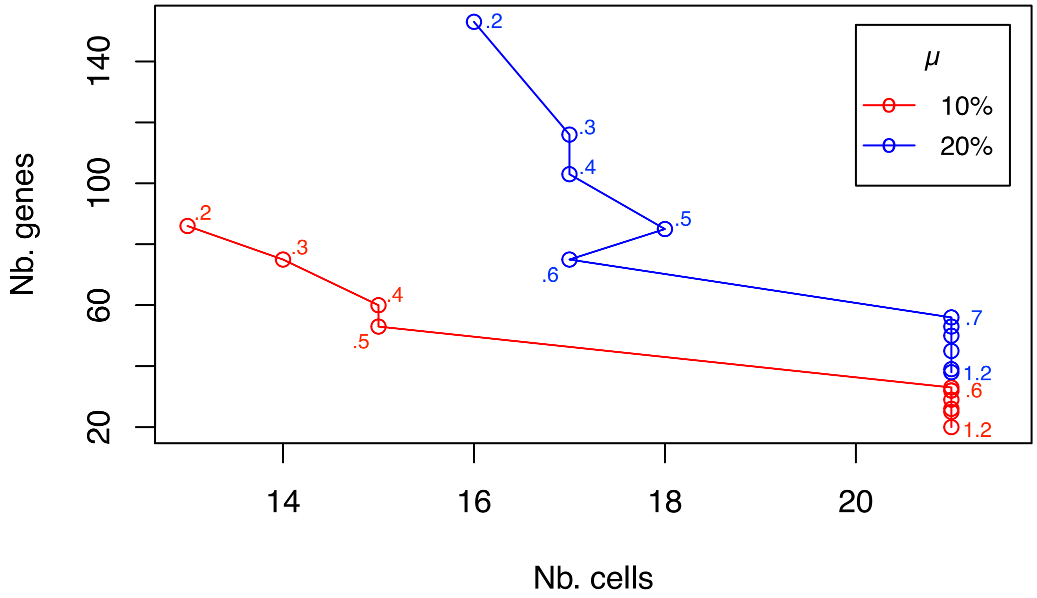

Figure 4: Number of cells and genes identified by MicroCellClust in the GARP+ Treg data in function of the κ parameter. The results are reported for two different values of the μ parameter.

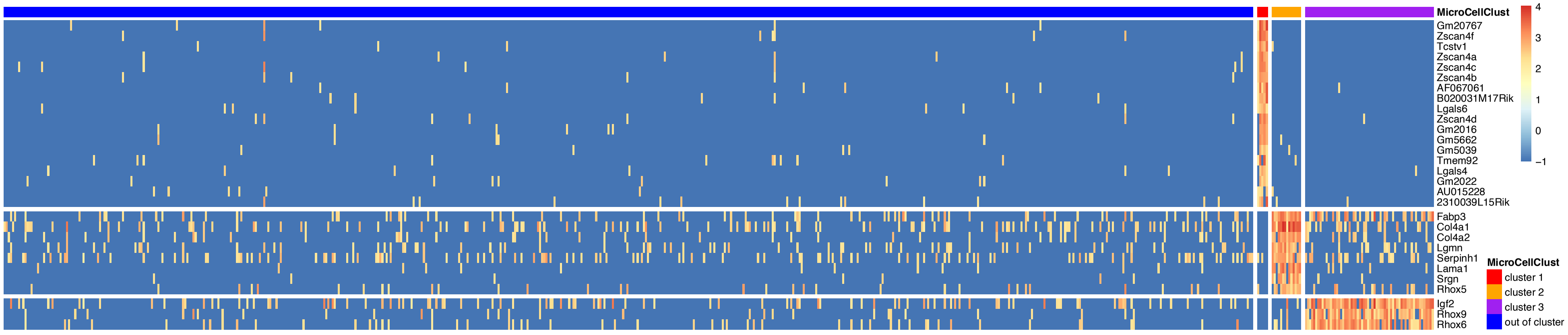

Figure 5: 3 subpopulations are identified by MicroCellClust in mouse embryonic stem cells. The reported biclusters are identified with $\kappa = 0.1$ and $\mu = 10\%$.

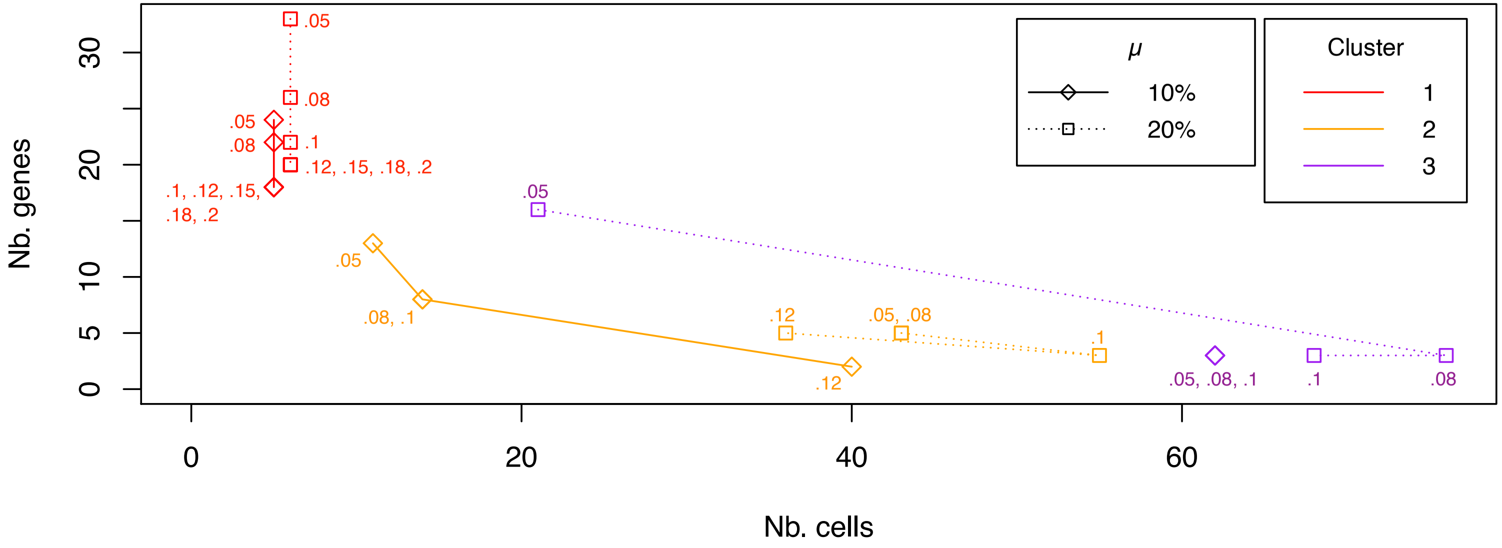

Figure 6: Number of cells and genes in function of the κ parameter for the 3 subpopulations indentified by MicroCellClust in the mouse stem cell data. The results are reported for two different values of the μ parameter.

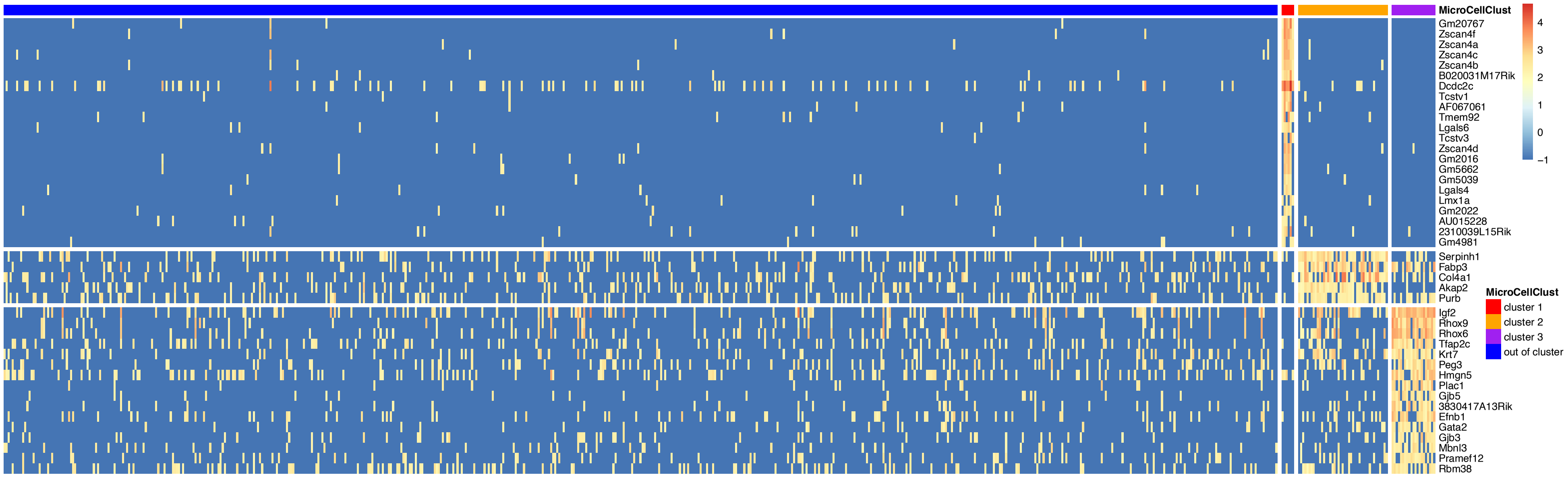

Figure 7: The 3 subpopulations identified in mouse embryonic stem cells when using $\mu = 20\%$. For clusters 2 and 3, $\kappa = 0.05$; for cluster 1 the marker genes identified with $\kappa = 1$ are reported (which is a subset of the set obtained with $\kappa = 0.05$).

C Gene Ontology enrichment analysis

We report the results of the Gene Ontology enrichment analysis for the two subpopulations identified by MicroCellClust from the breast cancer sample studied in sections 3.3 and 3.4 of the main manuscript. This analysis has been performed using the `clusterProfiler` R package [3]. For each one, the 20 most significantly enriched GO terms are reported. A functional interpretation of these terms is included in the main manuscript.

C.1 GO functions for the Treg related subpopulation within CD4 T cells

	ID	Description	p.adjust	GeneRatio
1	GO:0042110	T cell activation	3.1138e-09	10/20
2	GO:0050863	regulation of T cell activation	3.1138e-09	9/20
3	GO:0051249	regulation of lymphocyte activation	3.1138e-09	10/20
4	GO:0007159	leukocyte cell-cell adhesion	1.3524e-07	8/20
5	GO:1903037	regulation of leukocyte cell-cell adhesion	1.8311e-06	7/20
6	GO:0042098	T cell proliferation	2.1847e-06	6/20
7	GO:0045589	regulation of regulatory T cell differentiation	2.1847e-06	4/20
8	GO:0051251	positive regulation of lymphocyte activation	2.1847e-06	7/20
9	GO:0045066	regulatory T cell differentiation	2.4163e-06	4/20
10	GO:0050870	positive regulation of T cell activation	2.5437e-06	6/20
11	GO:0050670	regulation of lymphocyte proliferation	2.5953e-06	6/20
12	GO:0032944	regulation of mononuclear cell proliferation	2.5953e-06	6/20
13	GO:1903039	positive regulation of leukocyte cell-cell adhesion	2.9698e-06	6/20
14	GO:0002696	positive regulation of leukocyte activation	2.9698e-06	7/20
15	GO:0070663	regulation of leukocyte proliferation	2.9698e-06	6/20
16	GO:0050867	positive regulation of cell activation	3.3784e-06	7/20
17	GO:0022407	regulation of cell-cell adhesion	3.5021e-06	7/20
18	GO:0045785	positive regulation of cell adhesion	3.5021e-06	7/20
19	GO:0002517	T cell tolerance induction	4.5396e-06	3/20
20	GO:0022409	positive regulation of cell-cell adhesion	5.0522e-06	6/20

C.2 GO functions for the cell-cycle subpopulation in GARP+ Tregs

	ID	Description	p.adjust	GeneRatio
1	GO:0140014	mitotic nuclear division	1.6551e-10	11/31
2	GO:0000280	nuclear division	3.2562e-10	12/31
3	GO:0048285	organelle fission	6.8814e-10	12/31
4	GO:0006260	DNA replication	2.2744e-09	10/31
5	GO:0000070	mitotic sister chromatid segregation	6.0179e-07	7/31
6	GO:0030261	chromosome condensation	1.4243e-06	5/31
7	GO:0007076	mitotic chromosome condensation	1.4243e-06	4/31
8	GO:0000819	sister chromatid segregation	1.7845e-06	7/31
9	GO:0007059	chromosome segregation	2.9742e-06	8/31
10	GO:0000075	cell cycle checkpoint	3.5763e-06	7/31
11	GO:0031572	G2 DNA damage checkpoint	1.1120e-05	4/31
12	GO:0098813	nuclear chromosome segregation	1.1120e-05	7/31
13	GO:0071103	DNA conformation change	4.5714e-05	7/31
14	GO:0006323	DNA packaging	5.3637e-05	6/31
15	GO:0045787	positive regulation of cell cycle	1.2577e-04	7/31
16	GO:0044839	cell cycle G2/M phase transition	1.8450e-04	6/31
17	GO:0031570	DNA integrity checkpoint	2.2603e-04	5/31
18	GO:0007088	regulation of mitotic nuclear division	2.6408e-04	5/31
19	GO:0006310	DNA recombination	2.6518e-05	6/31
20	GO:1901987	regulation of cell cycle phase transition	3.7422e-04	7/31

D MicroCellClust implementation

The constrained optimization problem solved by MicroCellClust is formulated below, as is the main manuscript:

$$(I^*, J^*) = \underset{\substack{I \subseteq \mathcal{G} \\ J \subseteq \mathcal{C}}}{\operatorname{argmax}} \sum_{i \in I} \Big(\sum_{j \in J} m_{ij} - \kappa \sum_{k \in \mathcal{C} \setminus J} \max\{0, m_{ik}\} \Big) \tag{1}$$

$$\text{such that} \quad \frac{\Big| \big\{ (i,j) \,\big|\, i \in I, j \in J, m_{ij} < 0 \big\} \Big|}{|I| \cdot |J|} \leq \mu \tag{2}$$

D.1 Constraints

Equation (2) introduces a constraint on the proportion of negative values allowed in the bicluster. In our implementation, we represent this generic constraint by a global constraint and an additional constraint on each gene:

$$-\sum_{i \in I} \sum_{j \in J} \min\{0, \operatorname{sign}(m_{ij})\} \leq \mu |I||J| \tag{3}$$

$$-\sum_{j \in J} \min\{0, \operatorname{sign}(m_{ij})\} \leq \mu |J| \qquad \forall i \in I \tag{4}$$

The global constraint (3) guarantees that at most μ% of negative values are included inside the selected bicluster. The additional constraints (4) make sure that such a property is also satisfied for each selected gene. One could define different μ values for constraints (3) and (4), and typically be more permissive (a larger μ) for individual genes than for the selected bicluster overall. Our current implementation uses a common μ value (by default, $\mu = 10\%$) which produces good results in our experiments.

D.2 Heuristic search evaluation

The MicroCellClust implementation is inspired from the CPGC algorithm [4] used to solve the max-sum submatrix problem. Nevertheless, the upper bound used by CPGC is not as effective at pruning the search tree because of the adapted objective function defined by equation (1) and the additional constraints above. This results from the fact that the constrained optimization problem is designed here to search for biclusters with a *small* number of cells while the original CPGC objective could lead to include many cells. Consequently, there is often an important gap between the actual objective value in (1) and this (optimistic) upper bound. Heuristics are therefore used to speed up the search procedure.

More specifically, the search space of MicroCellClust contains $2^{|\mathcal{C}|}$ possible cell subsets to consider. Indeed, for any specific subset of cells, the associated marker genes to be included in an optimal bicluster can be found in linear time. This is referred to as an implicit search space in the main manuscript. Our implementation of MicroCellClust includes a heuristic search that only evaluates a fraction of the possible cell subsets. At each level of the breadth-first search, only the t subsets of cells with highest objective values are kept. The solver further considers only supersets of these cell subsets at the next level. Such a heuristic search is not guaranteed to return an optimal solution. However an optimal solution can be found with high probability whenever t is appropriately chosen. Indeed, the distribution of objective values at a given level roughly follows a power law and t is chosen so as to ignore the long tail of the distribution (see figures 8 and 10). We report here comparative results with an exact search strategy (branch-and-bound [5]) to support this claim.

The heuristic search is evaluated on the controlled experiment described in section 3.1 of the main manuscript. This experiment consists of 100 independent runs with $|\mathcal{C}| = 50$. The solver first evaluates all the biclusters corresponding to pairs of cells (*i.e.* level 2 in the search tree). Figure 8 shows that the distribution of objective values for these biclusters approximately follows a power law. A visual inspection of this plot illustrates that the ≈ 20 first biclusters have an objective value above average (past this point the slope of the distribution becomes small, corresponding to the long tail of the power law). It is assumed that at least one of these pairs must be included in the cell subset of the optimal solution. In our controlled experiment (see section 3.1 of the main manuscript), the heuristic search at level 2 with $t = 20$ produces the same results, for each of the 100 independent runs, as an exhaustive search. The same results are obtained when applying this heuristic at each search level. With a lower value such as $t = 10$, the optimum solution is missed for 15 out of 100 runs.

Figure 9 shows the number of visited nodes during branch-and-bound (exact search) and heuristic search for one run. The metaparameter μ is set here equal to 0%, which implies that such a (non negativity) constraint already eliminates a lot of candidate solutions. Yet, branch-and-bound still has an exponential complexity as a function of the number of cells. In contrast, the heuristic search has a polynomial complexity. Its computational bottleneck is the evaluation of all pairs at level 2, *i.e.* $\frac{|\mathcal{C}|^2 - |\mathcal{C}|}{2} \in \mathcal{O}\big(|\mathcal{C}|^2\big)$ (dashed line on Fig. 9).

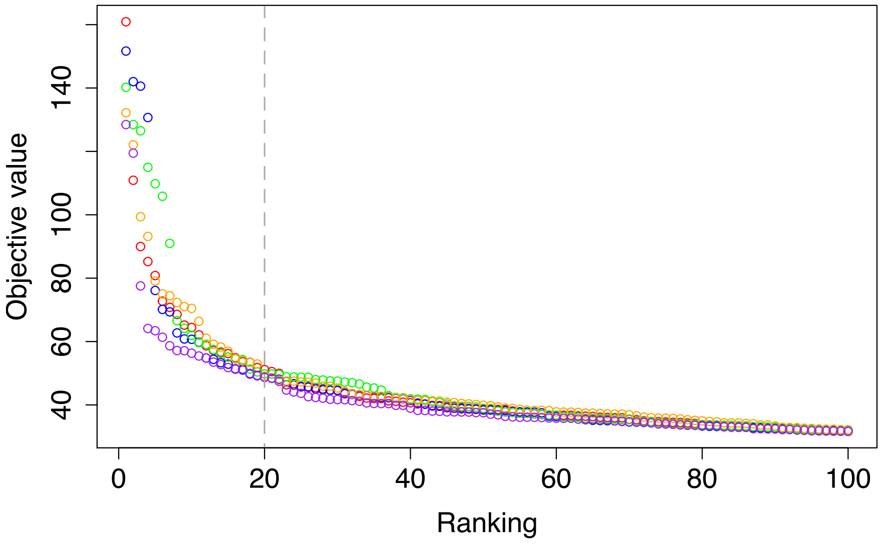

Figure 8: Distributions of the objective values of the 100 best pairs of cells for 5 runs of the GARP+ vs CD8+ controlled experiment. The distributions follow a power law the slope of which becomes small around rank 20.

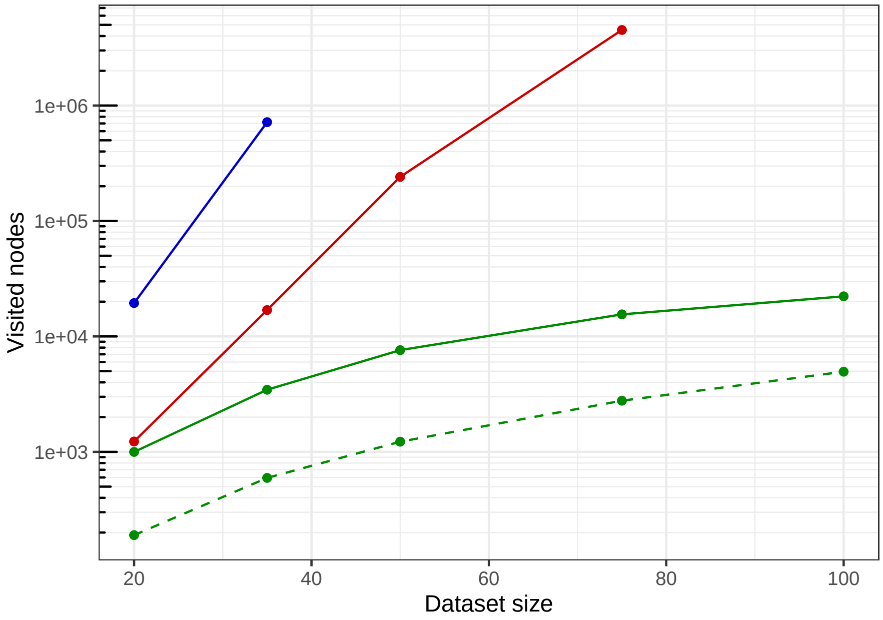

Figure 9: Number of biclusters (in logarithmic scale) evaluated for one of the runs of the controlled experiment with $\mu = 0\%$ during a full search **(blue)**, branch-and-bound **(red)**, and heuristic search with $t = 20$ **(green)** (the dashed line represents the number of pairs).

Dataset	Section	Figure	t
GARP+ vs CD8+	3.1	8	20
Jurkat vs 293T (100 cells)	3.2	10(a)	20
Jurkat vs 293T (1,000 cells)	3.2	10(b)	50
Breast cancer CD4+	3.3	10(c)	100
Breast cancer GARP+	3.4	10(d)	20
Mouse stem cell (top-1)	3.5	10(e)	20
Mouse stem cell (top-3)	3.5	10(f)	100

Table 2: Values for the heuristic parameter t for the experiments described in the main manuscript (the 2nd column refers to the related section). The 3rd column refers to the figures in the current document from which the values of t are inferred.

D.3 Values of the heuristic parameter t

Table 2 indicates the values of t that are chosen for the different datasets by observing the distributions of the objective values at level 2.

D.4 Execution time and memory analysis

The computational cost of the MicroCellClust solver is analyzed using the Jurkat versus 293T controlled experiment described in section 3.2 of the main manuscript. These experiments are executed on a MacBook Pro laptop.[1] The solver stops the search whenever no improvement of the current best objective value is found during, by default, 25 search levels after the best solution. Here, we consider datasets with 10% of rare cells, *i.e.* the worst case scenario in terms of execution time, and use $t = 20$ as parameter for the heuristic search.

Figure 11 shows that the execution time follows a quadratic distribution in terms of the number of cells. A quadratic regression is performed to infer the execution time for larger datasets. This model predicts an execution time of 22 minutes for a dataset of 1,500 cells. We measured 20.24 minutes when running such an instance of the Jurkat vs 293T data, confirming the quadratic assumption. The data reported in figure 11 also indicate that the memory load increases linearly with the number of cells[2] (the current version of the Scala code does not use a sparse representation of the data matrix).

E Additional results

E.1 Geneset reproducibility for Treg/CD8 experiment

Section 3.1 of the main manuscript illustrates that MicroCellClust achieves a high F_1-score at identifying multiple resamplings of a rare subpopulation of GARP+ Tregs among CD8 T cells. We report in figure 3 of the main manuscript a representative result: the 5 Tregs are perfectly identified, together with Treg related genes such as FOXP3, IL2RA and CCR8. These characteristic genes are also reidentified in the vast majority of the 100 runs performed in this experiment. Table 3 (a) shows that these genes, and several others, are present in nearly all of the 54 runs where MicroCellClust identifies the 5 Tregs perfectly. This illustrates the ability of MicroCellClust to reidentify important marker genes when resampling the data. A high occurrence is also observed when considering all the runs (table 3 (b)), thus including those where only a subset of the Tregs form the solution and/or some CD8 T cells are also selected.

E.2 Max-sum submatrix result

Figure 12 illustrates the result of the max-sum submatrix problem on an instance of the GARP+ versus CD8+ controlled experiment (section 3.1 of the main manuscript). The identified submatrix is composed of 42 cells (*i.e.* 84% of the cells) and 1,879 genes which contribute positively to the overall sum. Such a large bicluster is due to the objective function of the max-sum submatrix problem which does not focus on identifying *rare* subsets of cells, contrary to the MicroCellClust objective.

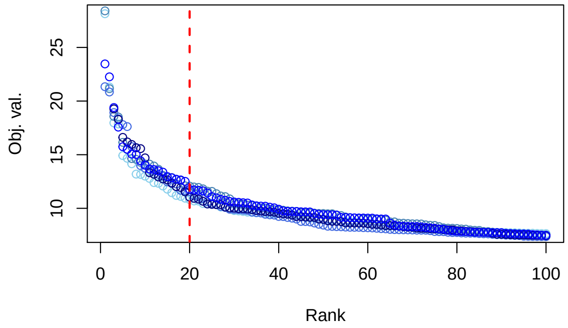

(a) 5 runs of the Jurkat vs 293T experiment with 10 rare cells among total of 100 cells ($\kappa = 1$).

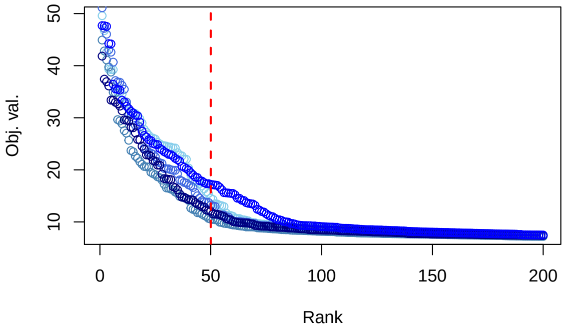

(b) 5 runs of the Jurkat vs 293T experiment with 10 rare cells among total of 1,000 cells ($\kappa = 0.1$).

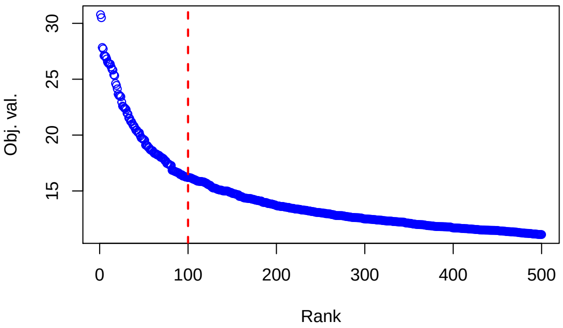

(c) Breast cancer CD4+ ($\kappa = 0.5$).

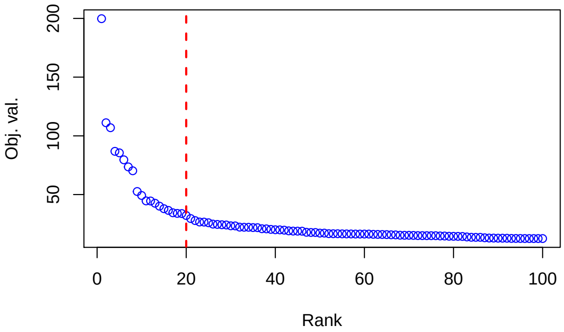

(d) Breast cancer GARP+ ($\kappa = 0.6$).

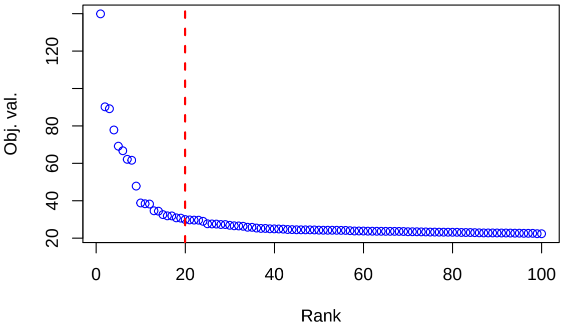

(e) Mouse stem cell ($\kappa = 0.1$).

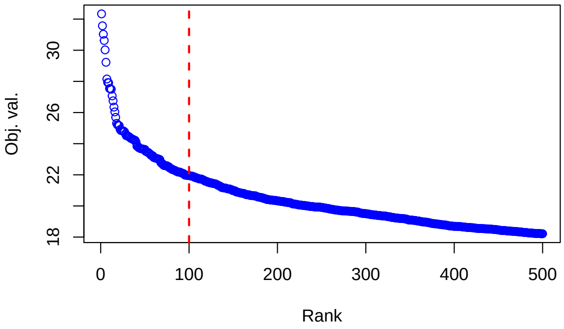

(f) Mouse stem cell ($\kappa = 0.1$) after removing the cells of the top-1 result. This has a large impact on the shape of the distribution, and motivates a higher value of $t = 100$ to find the two next biclusters.

Figure 10: Distributions of the objective values at level 2. The dashed red lines indicate the chosen values for the heuristic parameter t, *i.e.* the rank where the long tail starts.

Observed execution time and memory load:

Nb. cells	Exe. time [min]	Memory [MB]
50	0.03	140
100	0.16	180
200	0.49	333
500	2.50	705
1,000	9.87	1770

Execution time inferred by quadratic regression:

Nb. cells	Exe. time
1,500	22 min
2,000	39 min
2,500	1 h
5,000	4 h
10,000	16 h
20,000	5 days

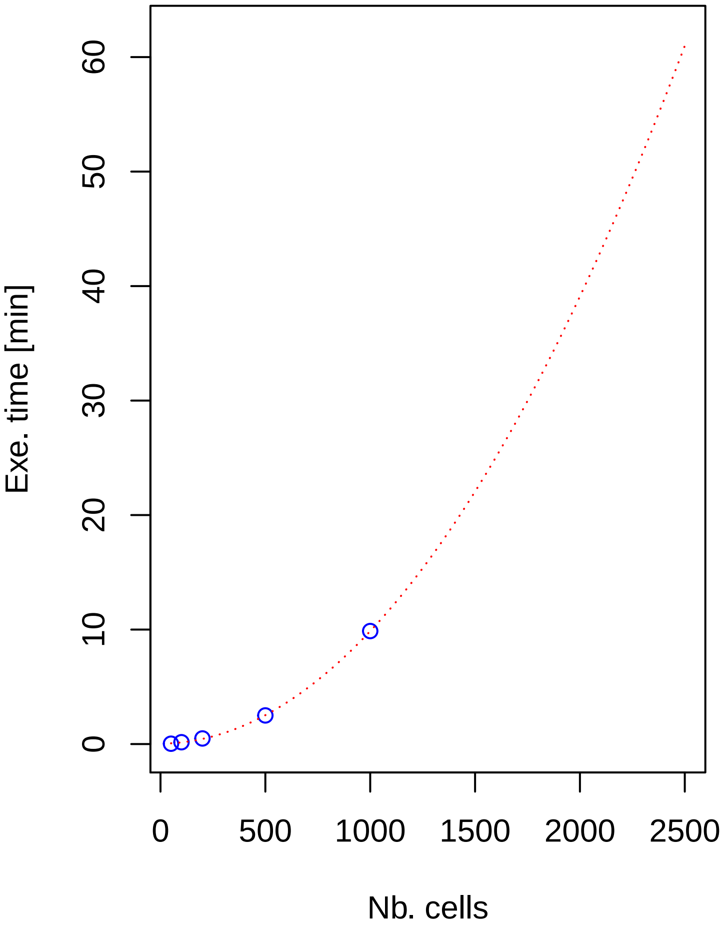

Figure 11: Observed execution time (average over 10 runs) and memory load during the Jurkat vs 293T experiment (with 10% of rare cells). The evolution of execution time is represented graphically (blue dots) with a quadratic regression (dashed red line).

Rank	Gene	Occurrence [%]
1	CD4	96
1	FOXP3	96
1	CTLA4	96
4	CCR8	94
5	TNFRSF18	87
6	IL2RA	85
7	CCDC22	81
7	DUSP4	81
9	AL137186.1	78
9	ICOS	78
11	IL1R2	74
12	HNRNPA1P21	67
13	ENTPD1	65
14	LINC02099	59
15	VDR	50
16	ENTPD1-AS1	48
17	F5	46
18	PMAIP1	44
19	TBC1D4	39
20	DNPH1	37

(a) Occurrence among perfect runs

Rank	Gene	Occurrence [%]
1	CTLA4	85
2	FOXP3	84
3	CCR8	83
4	CD4	80
4	TNFRSF18	80
6	IL2RA	76
7	DUSP4	72
8	CCDC22	69
9	AL137186.1	68
10	IL1R2	64
10	ICOS	64
12	ENTPD1	61
13	HNRNPA1P21	59
14	VDR	55
15	LINC02099	51
16	ENTPD1-AS1	49
17	F5	44
18	TBC1D4	37
19	TNFRSF4	34
20	DNPH1	33

(b) Occurrence among all runs

Table 3: Occurrence of the 20 most recurring genes identified by MicroCellClust among **(a)** only the 54 runs with perfect precision and recall (*i.e.* the 5 Tregs form exactly the solution), and **(b)** all the 100 runs.

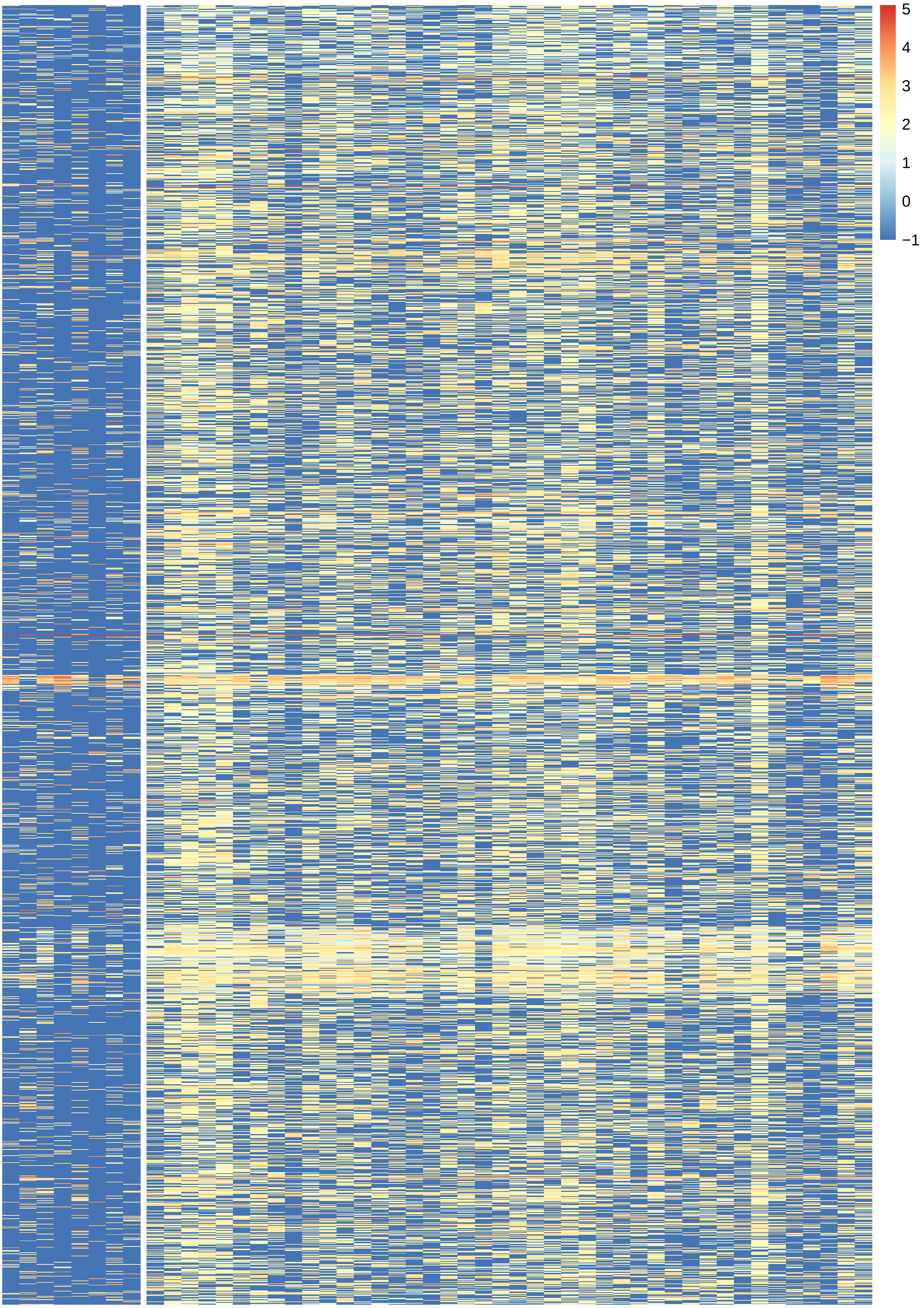

Figure 12: Submatrix of maximal sum for a dataset from the GARP+ vs CD8+ experiment. The bicluster is composed of 42 cells (on the right) and 1,879 genes.

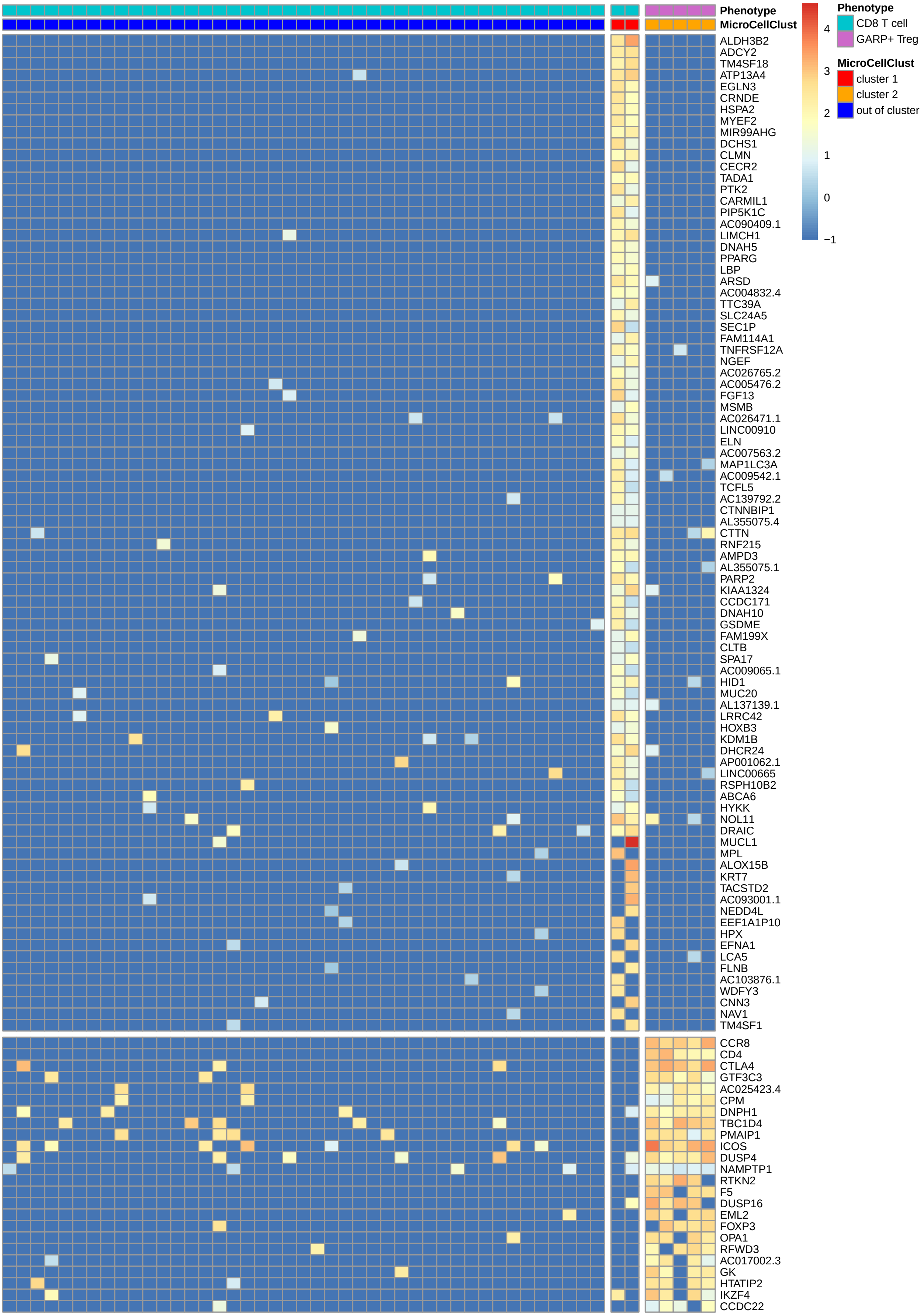

Figure 13: Top-2 result of a run where MicroCellClust first identifies 2 CD8 T cells as the first solution. The 5 GARP+ Tregs are identified in second place.

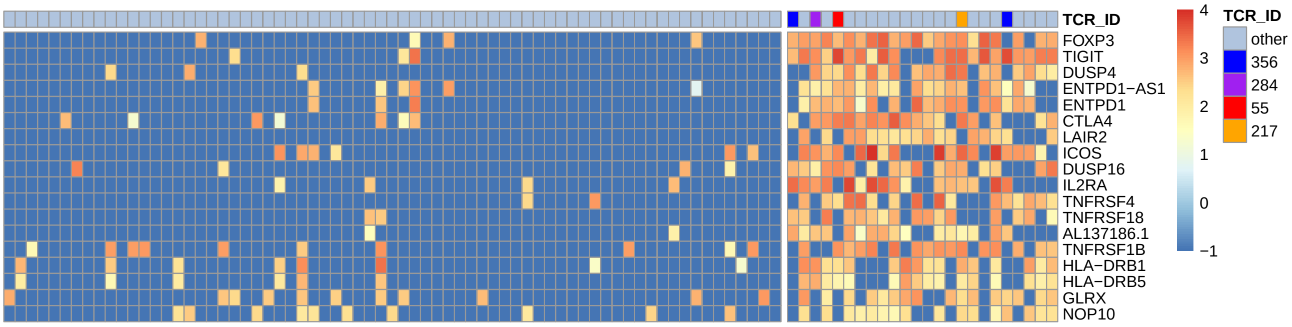

Figure 14: The Treg related subpopulation is also identified withing the CD4 T cells when running MicroCellClust only on the tumor cells.

E.3 Illustration of top-2 result in Treg/CD8 experiment

During the GARP+ versus CD8+ experiment (see section 3.1 in the main manuscript), MicroCellClust identifies a GARP+ Treg related bicluster in 81 of the 100 independent runs (71 of them with a 100% precision). In the remaining 19 runs, MicroCellClust returns a subset of the CD8 T cells. In these cases, a Treg related solution is obtained when searching for the top-2 solution, *i.e.* running again MicroCellClust while excluding the previously identified cells from the search.

Figure 13 illustrates such a case. Two CD8 T cells are identified as the first cluster as they coexpress a large number of genes that are very lowly expressed in the other cells. In this instance, the objective value of this bicluster is higher than the one of the cluster formed by the 5 GARP+ Tregs and corresponding marker genes. In general, the top-k strategy can be used to identify different (mutually exclusive) subpopulations of cells in the data.

E.4 Identification of CD4 subpopulation among tumor cells only

In section 3.3 of the main manuscript, MicroCellClust is used to identify a subpopulation within CD4 T cells originating from both tumor and healthy breast cells. It turns out that the identified subpopulation, which can be linked to Tregs, is originating specifically from the tumor cells. Therefore it can also be identified when running MicroCellClust on the tumor cells only (and appropriately tuning κ to 0.7) as illustrated in figure 14, although it no longer consists in a rare subpopulation ($\approx$ 25% of the cells). The cutoff value when filtering genes must therefore be raised to 30% to identify genes such FOXP3, which are no longer characteristic of a rare subpopulation.

References

[1] Simone Picelli, Omid R. Faridani, Åsa K Björklund, Gösta Winberg, Sven Sagasser, and Rickard Sandberg. Full-length RNA-seq from single cells using Smart-seq2. *Nature Protocols*, 9:171–181, 2014.

[2] Orian Bricard. Index hopping correction for scRNA-seq. https://rdrr.io/github/obricard/cleanhop/.

[3] Guangchuang Yu, Li-Gen Wang, Yanyan Han, and Qing-Yu He. clusterProfiler: an R package for comparing biological themes among gene clusters. *Omics: a journal of integrative biology*, 16(5):284–287, 2012.

[4] Vincent Branders, Pierre Schaus, and Pierre Dupont. Combinatorial optimization algorithms to mine a sub-matrix of maximal sum. In Annalisa Appice, Corrado Loglisci, Giuseppe Manco, Elio Masciari, and Zbigniew W. Ras, editors, *New Frontiers in Mining Complex Patterns*, pages 65–79. Springer International Publishing, Cham, 2018.

[5] Ailsa H. Land and Alison G. Doig. An automatic method of solving discrete programming problems. *Econometrica*, 18(3):497–520, 1960.

[6] David B. Dahl. Integration of R and Scala using rscala. *Journal of Statistical Software*, 92(4):1–18, 2020.

[1] Mac OS 10.15.7; 2.7 GHz Intel Core i7 CPU; 16 GB RAM.

[2] The table reports the memory consumption of the Scala code. The R code executes the former using the `rscala` library [6], which uses $\approx$ 240 MB.
